# RETRACTION: Application of Hyperbaric Oxygen Therapy in Diabetic Foot Ulcers: A Meta‐Analysis

**DOI:** 10.1111/iwj.70437

**Published:** 2025-03-26

## Abstract

**RETRACTION:** ChenH.‐R. , LuS.‐J. , WangQ. , LiM.‐L. , ChenX.‐C. , and PanB.‐Y. , “Application of Hyperbaric Oxygen Therapy in Diabetic Foot Ulcers: A Meta‐Analysis,” International Wound Journal 21, no. 42024): e14621, 10.1111/iwj.14621.38531355 PMC10965274

The above article, published online on 26 March 2024, in Wiley Online Library (http://onlinelibrary.wiley.com/), has been retracted by agreement between the journal Editor in Chief, Professor Keith Harding; and John Wiley & Sons Ltd. Following an investigation by the publisher, all parties have concluded that this article was accepted solely on the basis of a compromised peer review process. The editors have therefore decided to retract the article. The authors did not respond to our notice regarding the retraction.

**RETRACTION:** H.‐R. Chen , S.‐J. Lu , Q. Wang , M.‐L. Li , X.‐C. Chen , and B.‐Y. Pan , “Application of Hyperbaric Oxygen Therapy in Diabetic Foot Ulcers: A Meta‐Analysis,” International Wound Journal 21, no. 42024): e14621, 10.1111/iwj.14621.38531355 PMC10965274

The above article, published online on 26 March 2024, in Wiley Online Library (http://onlinelibrary.wiley.com/), has been retracted by agreement between the journal Editor in Chief, Professor Keith Harding; and John Wiley & Sons Ltd. Following an investigation by the publisher, all parties have concluded that this article was accepted solely on the basis of a compromised peer review process. The editors have therefore decided to retract the article. The authors did not respond to our notice regarding the retraction.

